# Identification and validation of a glycolysis-associated multiomics prognostic model for hepatocellular carcinoma

**DOI:** 10.18632/aging.202613

**Published:** 2021-03-03

**Authors:** Tuo Deng, Qian Ye, Chen Jin, Mingliang Wu, Kaiyu Chen, Jinhuan Yang, Ziyan Chen, XiXiang Yu, Gang Chen, Yi Wang

**Affiliations:** 1 Department of Hepatobiliary Surgery, The First Affiliated Hospital of Wenzhou Medical University, Wenzhou, China; 2 Key Laboratory of Diagnosis and Treatment of Severe Hepato-Pancreatic Diseases of Zhejiang Province, The First Affiliated Hospital of Wenzhou Medical University, Wenzhou, Zhejiang, China; 3 Department of Clinical Laboratory, Wenzhou People's Hospital, The Third Clinical Institute Affiliated to Wenzhou Medical University, Wenzhou, China; 4 Department of Epidemiology and Biostatistics, School of Public Health and Management, Wenzhou Medical University, Wenzhou, Zhejiang, China; 5 Department of Oncology, The First School of Clinical Medicine, Wenzhou Medical University, Wenzhou, Zhejiang, China

**Keywords:** hepatocellular carcinoma, glycolysis, prognostic signature, multiomic analysis, risk stratification

## Abstract

Increased glycolysis has been reported as a major metabolic hallmark in many cancers, and is closely related to malignant behavior of tumors. However, the potential mechanism of glycolysis in hepatocellular carcinoma (HCC) and its prognostic value are not well understood. To address this, we investigated glycolysis-related gene expression data of patients with HCC from TCGA and ICGC. Patients were categorized into three different glycolysis-associated subgroups: Glycolysis-M, Glycolysis-H, and Glycolysis-L. We found that Glycolysis-H combined with Glycolysis-M (Glycolysis-H+M) subgroup was associated with poor overall survival and distinct cancer stem cell characteristics and immune infiltrate patterns. Additionally, multiomics-based analyses were conducted to evaluate genomic patterns of glycolysis subgroups, including their gene mutations, copy number variations, and RNA-sequencing data. Finally, a glycolysis-associated multiomics prognostic model (GMPM) consisting of 19 glycolysis-associated genes was developed. The capability of GMPM in categorizing patients with HCC into high- and low-risk groups was validated with independent HCC datasets. Finally, GMPM was confirmed as an independent risk factor for the prognosis of patients with HCC. We believe that our findings provide new insights into the mechanism of glycolysis and highlight the potential clinical value of GMPM in predicting the prognosis of patients with HCC.

## INTRODUCTION

Hepatocellular carcinoma (HCC) is among the most common malignancies worldwide, and is associated with extremely high mortality with a rising trend [1]. Currently, the treatment options for patients with HCC mainly include partial hepatectomy, liver transplantation, systemic therapy, and interventional operations [2]. However, most of these treatments are limited to early-stage patients. Thus, there is a lack of effective therapies for patients in advanced stages, and this results in poor long-term outcomes for patients with HCC [3]. Moreover, clinicopathological features such as TNM staging can provide only ambiguous prognostic prediction abilities. Thus, there is an urgent need to develop an effective tool based on molecular biomarkers to identify and predict high-risk patients with HCC, who may have a poor prognosis.

The liver is a central metabolic coordinator that is specialized in regulating glucose metabolism [4]. One of the hallmarks of HCC cell metabolic aberrations is the increase in glycolysis rate with consequent lactate production, which is known as Warburg effect or aerobic glycolysis [5]. This phenomenon occurs even in the presence of mitochondria and oxygen [6]. It is widely observed that increased glycolysis is closely related to higher tumor invasion and proliferation as it provides energy to tumor cells [7]. Furthermore, interfering with metabolism of oncocytes promotes apoptosis by increasing the sensitivity to chemotherapeutic drugs, thereby suppressing tumorigenesis. This indicates that targeting glycolysis is a meaningful strategy in cancer treatment [8]. However, systematic investigation of the multiomics feature of glycolysis-associated molecules in predicting prognosis of patients with HCC is still insufficient. Therefore, exploring the relationship between glycolysis status and HCC development, and establishing a precise predictive model based on glycolysis-associated molecules, has the potential to improve personalized treatment design [9].

To address this gap in research, patients with HCC from The Cancer Genome Atlas (TCGA) were clustered into the following subgroups: Glycolysis-H, Glycolysis-M, and Glycolysis-L. Furthermore, the clinicopathological features were assessed, and their correlation with glycolysis status was investigated. We systematically explored the multiomics differences between the subgroups with respect to cancer stemness characteristics, immune infiltration states, somatic mutations, and copy number variations (CNVs). Importantly, we constructed a glycolysis-associated multiomics prognostic model and demonstrated its predictive performance.

## RESULTS

### Identification of HCC phenotype based on glycolysis-associated genes

**Figure 1:**
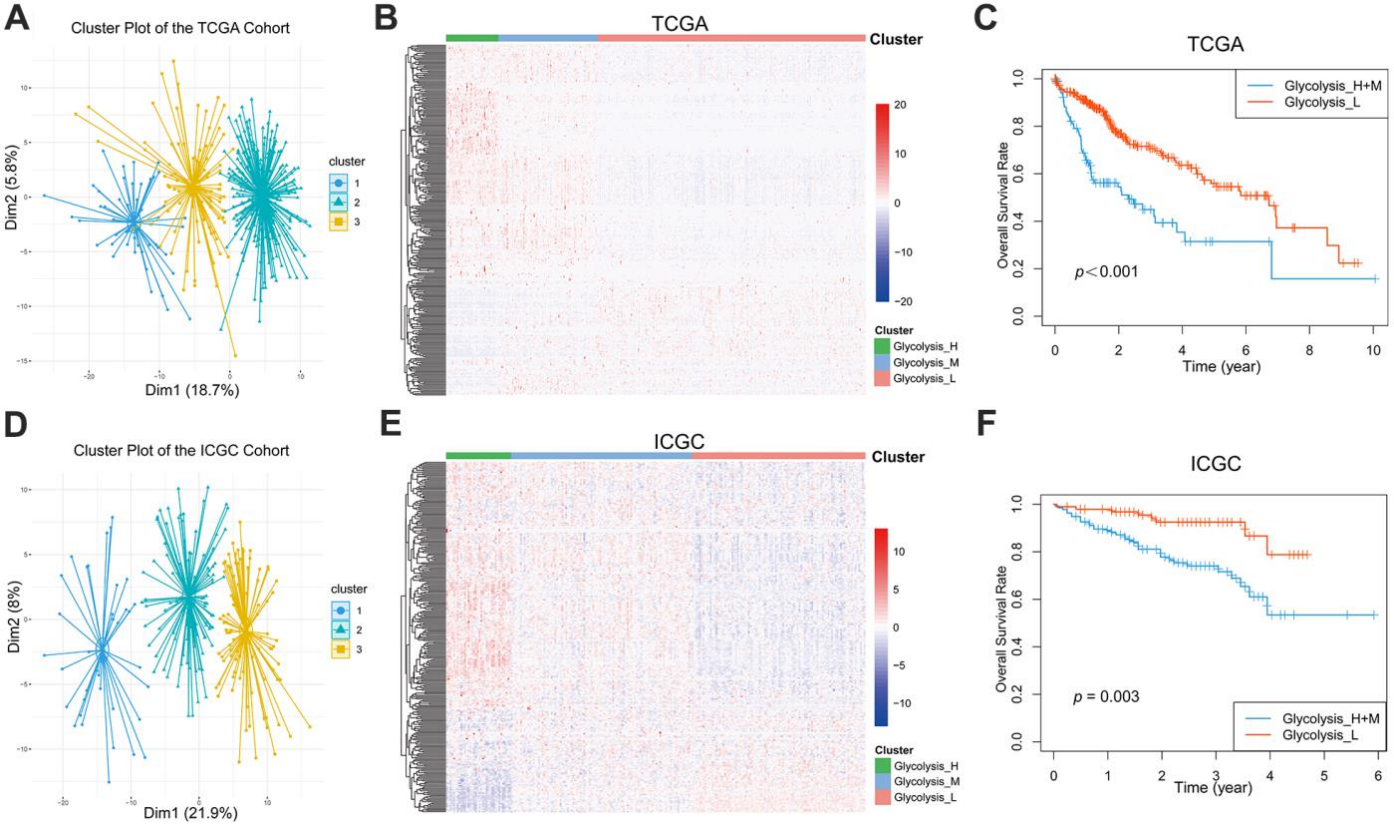
**Glycolysis-associated genes identified distinct HCC clusters with different OS.** (**A**, **B**) Three distinct clusters were generated by hierarchical clustering analysis based on the expression level of the 288 glycolysis-associated genes in the TCGA. (**D**, **E**) Three glycolysis-associated clusters were generated in the ICGC. (**C**, **F**) Kaplan-Meier survival curves of different glycolysis subtypes in the TCGA and ICGC. The Glycolysis-H+M subgroup had a worse OS than the Glycolysis-L subgroup (TCGA cohort: log-rank p<0.001; ICGC cohort: log-rank p= 0.003). HCC, hepatocellular carcinoma; OS, overall survival.

A total of 288 genes involved in glycolysis and glycolysis-related signaling pathways in KEGG were extracted (Supplementary Table 1). The expression profiles of these 288 glycolysis-associated genes were downloaded from TCGA-LIHC for hierarchical clustering analysis. According to elbow plot, 3 cluster stratification were adopted to classify patients from two independent cohort (Supplementary Figure 1). The analysis clustered the patients with HCC into three subgroups, Glycolysis-H, Glycolysis-M, and Glycolysis-L, on the basis of different expression patterns (Figure 1A, 1B). The Kaplan–Meier method was used to investigate the OS of the three glycolysis subgroups, and we observed that the subgroup Glycolysis-H combined with Glycolysis-M (Glycolysis-H+M) had worse OS than the Glycolysis-L subgroup (*p* < 0.001; Figure 1C).

We additionally extracted and analyzed the gene expression profiles of 288 glycolysis-associated genes from the LIRI-JP dataset in ICGC. Here as well, patients with HCC could be classified into three distinct glycolysis subgroups (Figure 1D, 1E). Further, the Kaplan–Meier plot showed a significant difference (*p* = 0.003) in the OS between the different glycolysis subgroups in the ICGC cohort as well (Figure 1F).

Furthermore, the clinicopathological features of these three glycolysis subgroups were compared, and the results confirmed significant differences in majority of the clinicopathological characteristics (Table 1). Patients in the Glycolysis-H and Glycolysis-M subgroups were associated with increased tumor grade (*p* < 0.001) and T-stage (*p* < 0.001).

**Table 1:** The comparison of clinical characteristics among different glycolysis subgroups of HCC patients in TCGA (n=363).

**Clinicopathological variables**	**Glycolysis-H (n = 15)**	**Glycolysis-M (n = 93)**	**Glycolysis-L (n = 255)**	***P*-value**
Age (years)				0.002
< 65	13 (86.7)	64 (69.6)	136 (53.3)	
≥ 65	2 (13.3)	28 (30.4)	119 (46.7)	
Gender				0.014
Male	5 (33.3)	65 (69.9)	176 (69.0)	
Female	10 (66.7)	28 (30.1)	79 (31.0)	
T-stage				< 0.001
T1+T2	8 (53.3)	56 (60.2)	204 (80.6)	
T3+T4	7 (46.7)	37 (39.8)	49 (19.4)	
N-stage				0.898
N0	11 (73.3)	62 (67.4)	173 (67.8)	
N1 + NX	4 (26.7)	30 (32.6)	82 (32.2)	
M-stage				0.234
M0	12 (80.0)	72 (77.4)	176 (69.0)	
M1 + MX	3 (20.0)	21 (22.6)	79 (31.0)	
AJCC stage				< 0.001
I + II	6 (42.9)	51 (58.6)	194 (81.5)	
III + IV	8 (57.1)	36 (41.4)	44 (18.5)	
AFP (ng/ml)				< 0.001
< 400	7 (87.5)	33 (51.6)	168 (84.0)	
≥ 400	1 (12.5)	31 (48.4)	32 (16.0)	
ECOG Performance Status				
0	4 (44.4)	26 (41.9)	132 (62.0)	< 0.001
1	1 (11.1)	15 (24.2)	65 (30.5)	
2	2 (22.2)	11 (17.7)	13 (6.1)	
3	1 (11.1)	8 (12.9)	3 (1.4)	
4	1 (11.1)	2 (3.2)	0	
Family history of cancer				0.058
No	12 (85.7)	57 (72.2)	135 (61.4)	
Yes	2 (14.3)	22 (27.8)	85 (38.6)	
Grade				< 0.001
G1-2	6 (42.9)	41 (44.1)	177 (70.5)	
G3-4	8 (57.1)	52 (55.9)	74 (29.5)	
Hepatitis C				0.967
No	13 (86.7)	80 (86.0)	217 (86.1)	
Yes	2 (13.3)	13 (14.0)	38 (14.9)	
Hepatitis B				0.042
No	15 (100)	70 (75.3)	181 (71.0)	
Yes	0	23 (24.6)	74 (29.0)	
Surgical margin resection status				0.002
R0	12 (85.7)	74 (80.4)	233 (93.2)	
Non-R0	2 (14.3)	18 (19.6)	17 (6.8)	
Vascular invasion				0.006
None	6 (66.7)	36 (51.4)	159 (69.7)	
Micro	3 (33.3)	25 (35.7)	62 (27.2)	
Macro	0	9 (12.9)	7 (3.1)	

### Cancer stem cell characteristics and immune infiltration evaluation in patients with different glycolysis subgroups

**Figure 2:**
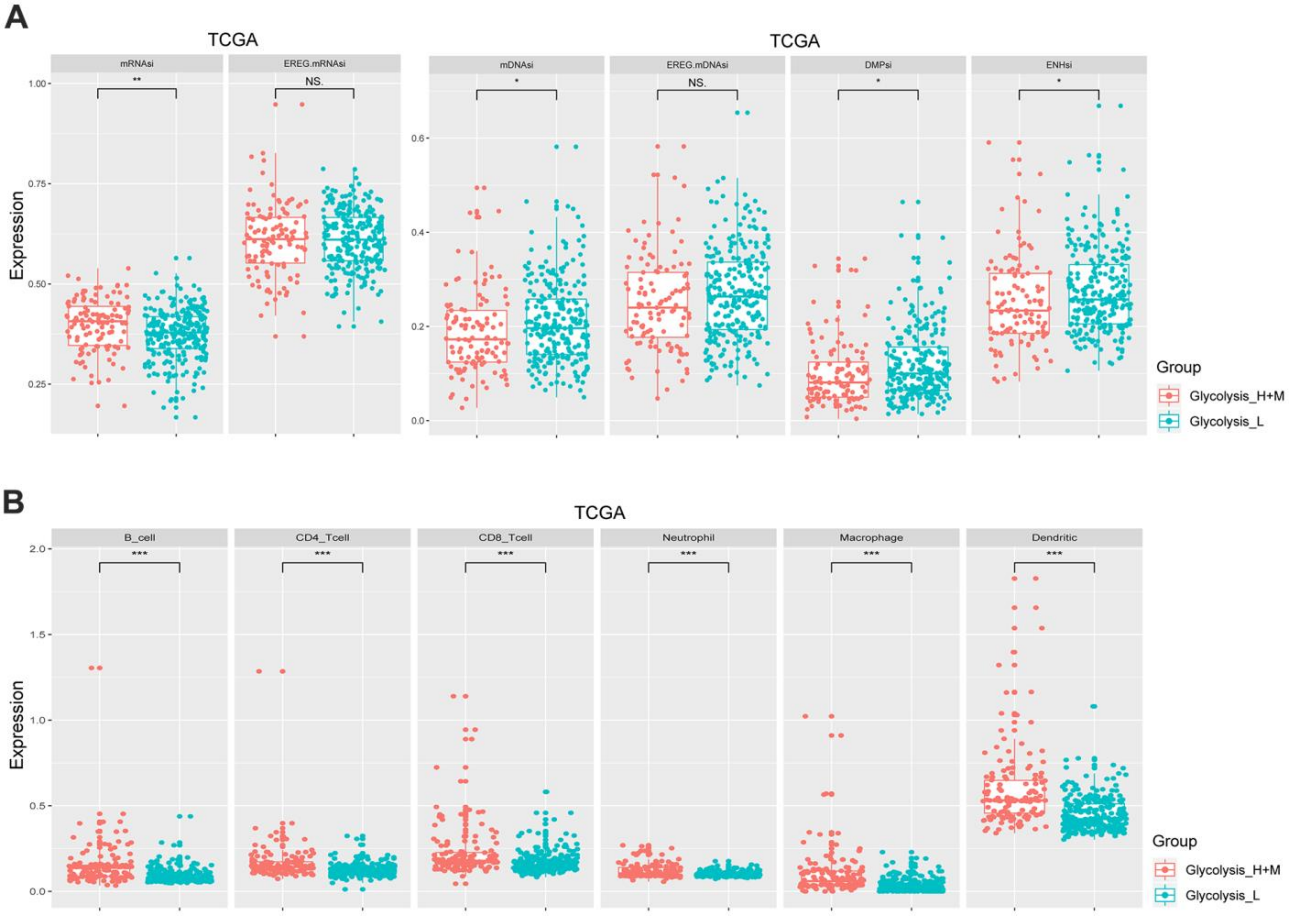
**Different glycolysis-associated HCC subtypes varied in cancer stemness and immune infiltration.** (**A**) Stemness indices of TCGA cohort. The Glycolysis-H+M subtype had a higher expression level of mRNAsi (p< 0.01), while the Glycolysis-L subtype had a higher expression level of mDNAsi (p< 0.05). (**B**) Immune cells with a significantly different proportion between Glycolysis-H+M and Glycolysis-L subgroups in the TCGA. *p< 0.05, **p< 0.01, ***p < 0.001.

mRNAsi and mDNAsi are the two main indices that reflect the stemness of samples based on gene expression and epigenetic features, respectively [10]. As shown in Figure 2A, the Glycolysis-H+M subgroup had a significantly higher stemness index in mRNAsi (*p* < 0.01) and, a lower epigenetic level (*p* < 0.05). The regulatory network of stem cell index and glycolysis-associated genes showed that mRNAsi negatively regulated *PFKFB3* and *VCAN*, while it positively regulated *CENPA*. Further, mDNAsi negatively regulated *QSX1*, *GPC4*, and *TGFA* (Supplementary Figure 2A).

The results of CIBERSORT showed significant differences in infiltrating immune cell types between the Glycolysis-H+M and Glycolysis-L subgroups including more abundant proportions of B cells, CD4+ and CD8+ T cells, neutrophils, macrophages, and dendritic cells in Glycolysis-H+M subgroup than the Glycolysis-L subgroup (*p* < 0.001; Figure 2B). Thus, Glycolysis-H+M subgroups showed a distinct immune infiltration pattern from Glycolysis-L subgroup. Moreover, in the regulatory network, macrophages and dendritic cells were closely related to the glycolysis-associated genes (Supplementary Figure 2B).

### Analysis of multiomics in patients with distinct glycolysis-associated subgroup

**Figure 3:**
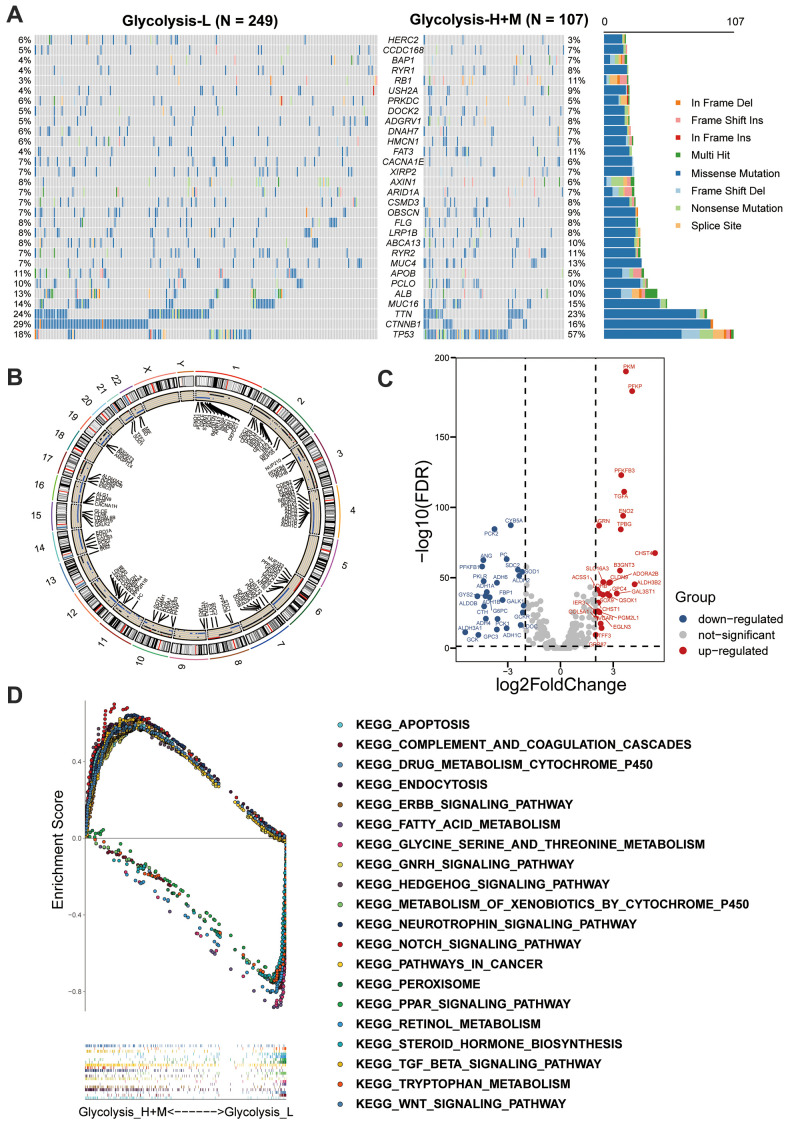
**Multi-omics analysis among glycolysis-associated HCC subgroups.** (**A**) The waterfall plot showed the mutation type of each patient and the proportion of mutation in each subgroup. 30 genes with the most frequent somatic mutation in HCC patients from TCGA were listed. (**B**) Differences in CNV profiles of the different glycolysis-associated HCC subtypes were visualized, gains of CNVs shown in black, and losses in blue. (**C**) Volcano plot showed the between Glycolysis-H+M subgroups and Glycolysis-L subgroup (FDR < 0.05 and |log2fold-change (FC)| > 1). (**D**) GSEA analysis of Glycolysis-H+M subgroups and Glycolysis-L subgroup. CNV, copy number variation; DEGs, differentially expressed genes; GSEA, Gene-set enrichment and functional enrichment analyses.

We further explored the glycolysis-associated HCC subgroups through multiomics profiles. The mutation profiles of patients with HCC were investigated, and the top 30 frequently somatically mutated genes in different glycolysis subgroups are presented in Figure 3A. Higher somatic mutation frequencies of *TP53* and *RB1* were observed in the Glycolysis-H+M subgroup. In contrast, somatic mutations in *CTNNB1* and *APOB* were significantly enriched in the Glycolysis-L subgroup (Figure 3A and Supplementary Figure 3).

Additionally, CNVs on chromosomal level were investigated, and their differences between the three glycolysis subgroups are displayed in Figure 3B. Glycolysis-H+M subgroup had CNVs on almost all chromosomes except sex chromosomes, and chromosomes 5 and 18. As illustrated, chromosomes 2, 3, 7, 10, 12, and 17 were mainly amplified, whereas chromosomes 4, 6, 11, 14, 15, 16, 21, and 22 were dominated by deletions.

Further, Volcano plot revealed 53 significant DEGs between the Glycolysis-H+M and Glycolysis-L subgroups, in which 27 genes were upregulated and 26 genes were downregulated (Figure 3C). GSEA-based KEGG analysis was used to investigate the underlying biological mechanisms related to the glycolysis-associated subgroups. The results revealed that “Notch signaling pathway,” “Wnt signaling pathway,” and “TGFβ signaling pathway” were enriched in the Glycolysis-H+M subgroup (Figure 3D).

### GSEA-based KEGG analysis and GO analysis

**Figure 4:**
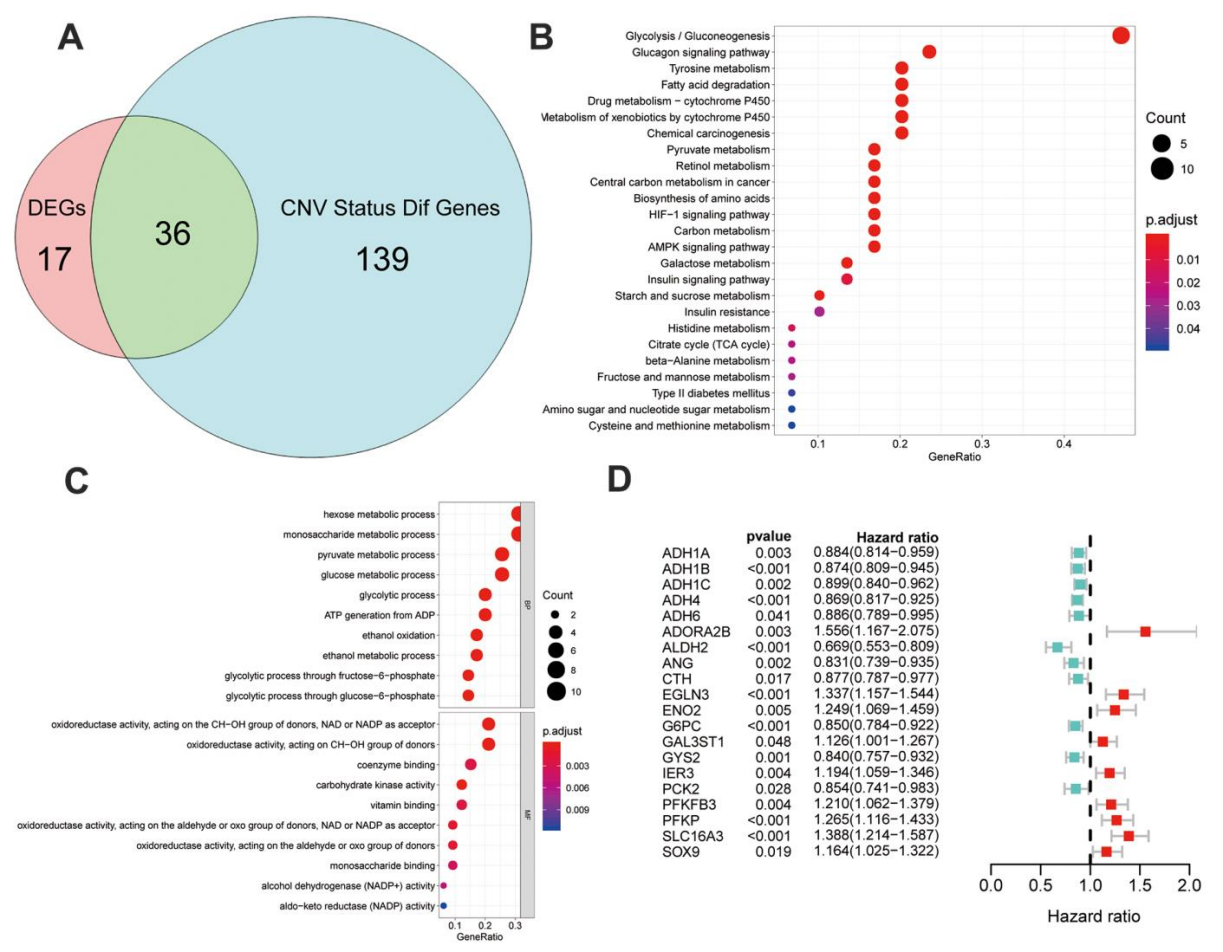
**Identification and analysis of different status genes among glycolysis subgroups based on multi-omics data.** (**A**) Venn diagram showed 36 overlapped genes in DEGs and genes with differently CNV status. (**B**) The results of gene ontology functions analysis of the 36 multi-omics based different status glycolysis genes. (**C**) KEGG pathways enrichment analysis of the 36 multi-omics based different status glycolysis genes. (**D**) 20 MOG-DEGs with a significant correlation with OS by univariable Cox analyses in the TCGA.

Intersection of 53 DEGs and 175 genes with different CNV statuses between the Glycolysis-H+M and Glycolysis-L subgroups in TCGA resulted in 36 multiomics glycolysis-associated differentially expressed genes (MOG-DEGs; Figure 4A).

KEGG-based pathway enrichment analysis of these 36 MOG-DEGs showed the highest enrichment in “glycolysis,” “gluconeogenesis,” “glucagon signaling pathway,” “tyrosine metabolism,” and “fatty acid degradation” (Figure 4B). “Glucose metabolism,” “oxidoreductase activity,” and “glycolysis/gluconeogenesis” were the most enriched terms in BP, MF, and CC, respectively (Figure 4C). These results clarified that these 36 MOG-DEGs were indeed involved in glycolysis.

To further validate the prognosis predictive potential of the MOG-DEGs in the OS of the patients with HCC, a univariable Cox regression analysis showed that 20 out of 36 MOG-DEGs had significant prognostic potential (*p* < 0.05; Figure 4D).

### Development and independent validation of GMPM

**Figure 5:**
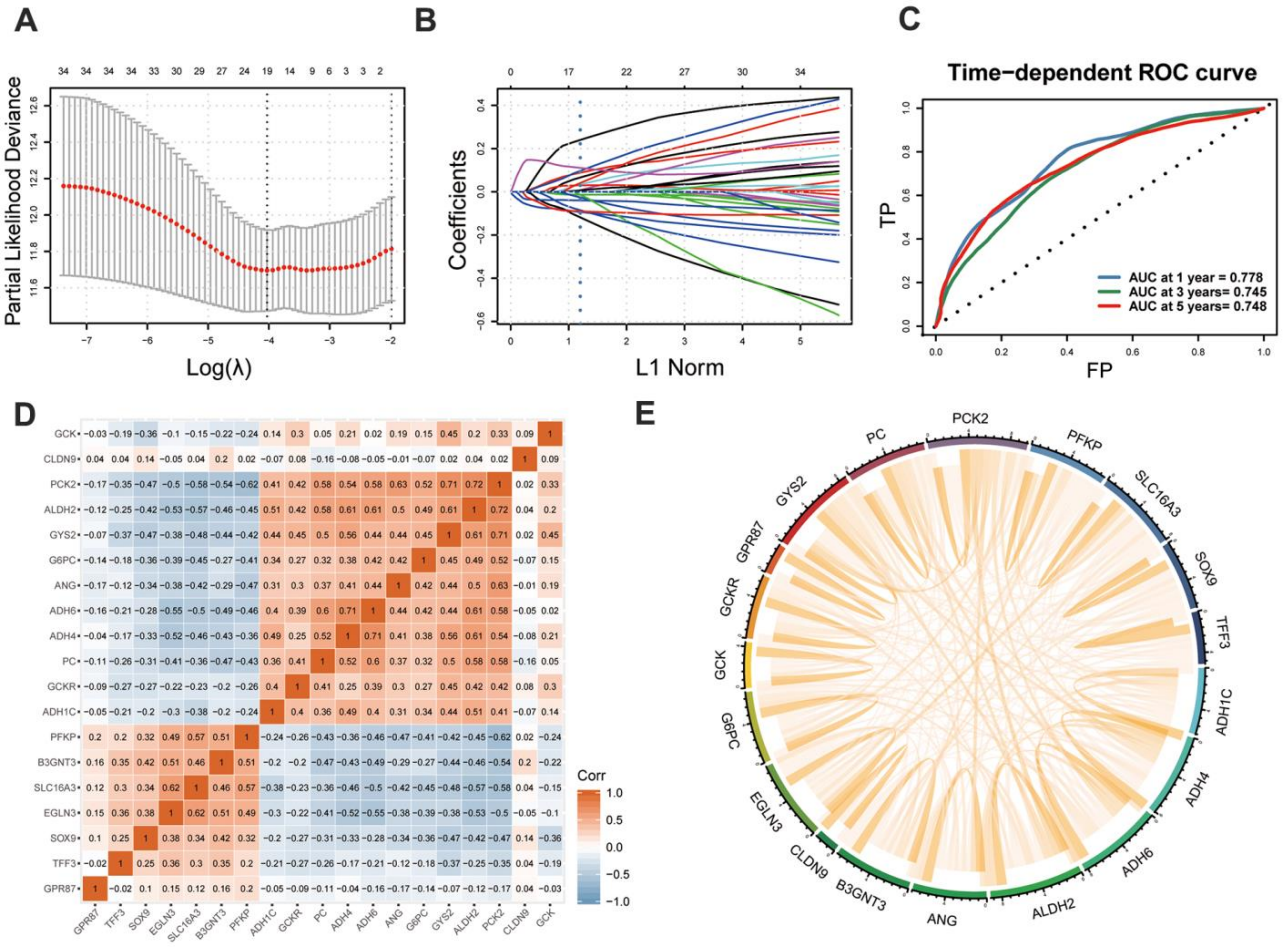
**Development of GMPM.** (**A**, **B**) LASSO regression analysis identified the 19 key MOG-DEGs in TCGA. (**C**) Time-dependent ROC curve analysis was performed to evaluate the diagnostic efficacy of GMPM. (**D**, **E**) The co-expression correlation between 19 key MOG-DEGs was showed. MOG-DEGs, multi-omics glycolysis-associated differentially expressed genes. ROC, receiver operating characteristic; GMPM, glycolysis-associated multi-omics prognostic model.

LASSO regression analysis screened out 19 MOG-DEGs with the most predictive potential from the 36 MOG-DEGs and constructed the GMPM (Figure 5A, 5B). A receiver operating characteristic curve showed that the area under the curve (AUC) of the GMPM signature in predicting the OS of patients with HCC at 1, 3, and 5 years was 0.778, 0.745, and 0.748, respectively (Figure 5C). We further investigated the coexpression levels of these 19 genes and found a weak to moderate correlation, where *PCK2* was significantly positively correlated with *ALDH2* and *GYS2* (*r* > 0.7; Figure 5D, 5E).

**Figure 6:**
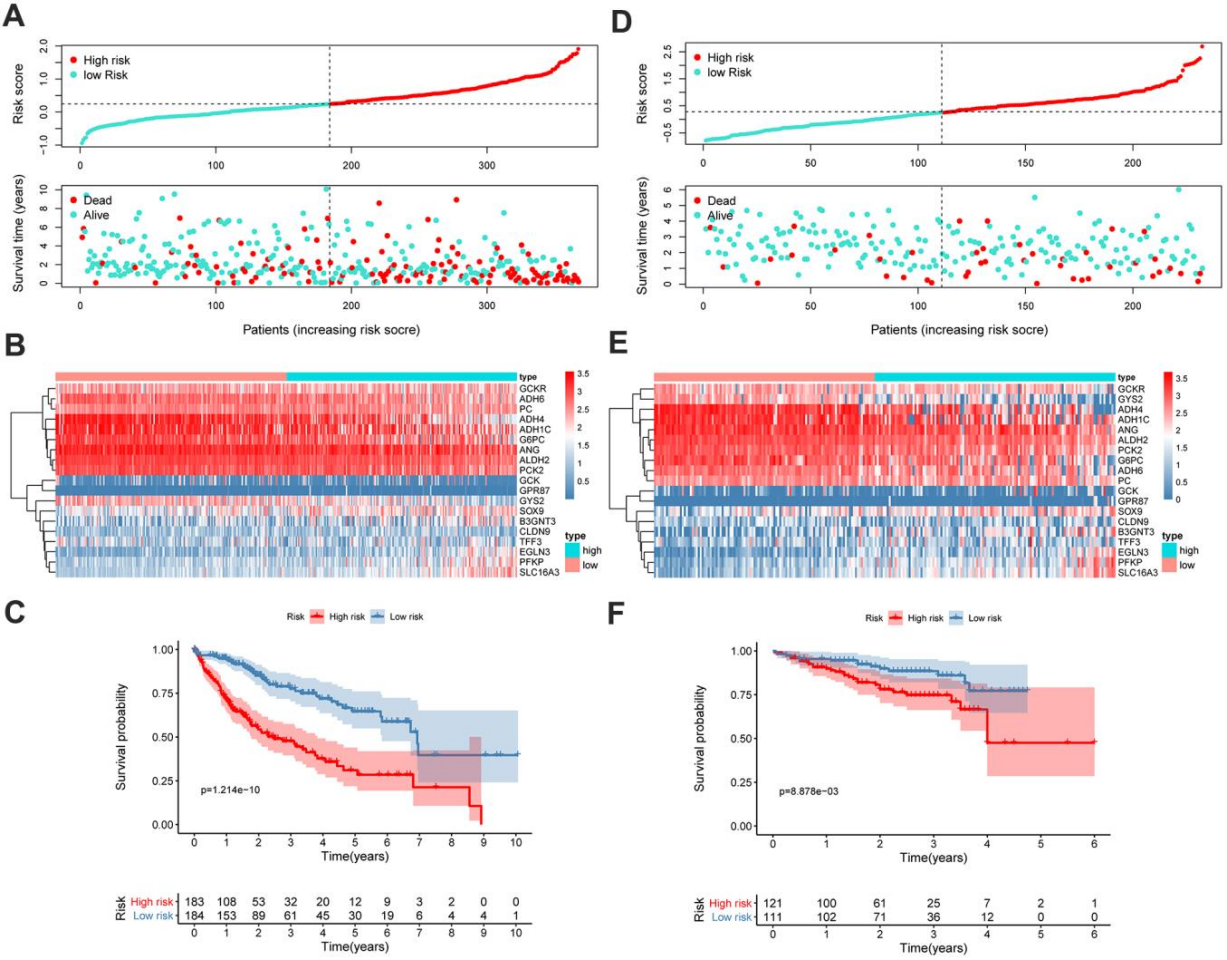
**GMPM predicts OS in HCC patients.** The distribution of survival status and risk scores in TCGA (**A**) and ICGC (**D**), respectively. The expression difference between high- and low-risk groups in TCGA (**B**) and ICGC (**E**). Kaplan-Meier survival curves to verify the predictive effect of GMPM in TCGA (**C**) and ICGC (**F**). GMPM, glycolysis-associated multi-omics prognostic model; OS, overall survival.

Using the GMPM signature and formula, the risk score of each patient in TCGA cohort was calculated. The coefficients of each gene in the GMPM are listed in Table 2. We used the median value (median = 0.28) of the risk score as the cutoff level to categorize the patients with HCC into high- and low-risk groups (Figure 6A). The relationship between the risk scores and OS was investigated, and the results indicated that patients in the high-risk group had a worse survival than those in the low-risk group (Figure 6C). The expression levels of *GCKR*, *ADH6*, *PC*, *ADH4*, *ADH1C*, *G6PC*, *ANG*, *ALDH2*, and *PCK2* were downregulated and those of *GCK*, *GPR87*, *GYS2*, *SOX9*, *B3GNT3*, *CLDN9*, *TFF3*, *EGLN3*, *PFKP*, and *SLC16A3* were upregulated in the high-risk group (Figure 6B).

**Table 2:** The coefficients of the risk score in GMPM.

**Gene**	**Coef**	**Gene**	**Coef**	**Gene**	**Coef**
ADH1C	-0.0136	EGLN3	0.117536	PC	0.284064
ADH4	-0.10821	G6PC	-0.04209	PCK2	0.102078
ADH6	0.067533	GCK	0.105903	PFKP	0.149495
ALDH2	-0.18404	GCKR	0.031161	SLC16A3	0.095468
ANG	-0.00241	GPR87	-0.09511	SOX9	0.019108
B3GNT3	-0.05977	GYS2	-0.12435	TFF3	-0.09511
CLDN9	0.043918				

We further validated and confirmed the prediction capability of the GMPM signature with the ICGC cohort. Using the same formula and cutoff value as that in TCGA cohort, the LIRI-JP cohort was also stratified into high- and low-risk groups. The results of the survival plot showed a poorer survival in the high-risk group than its counterpart, which was consistent with the results from TCGA cohort (Figure 6D–6F).

### GMPM risk score as an independent factor in predicting OS of patients with HCC

**Figure 7:**
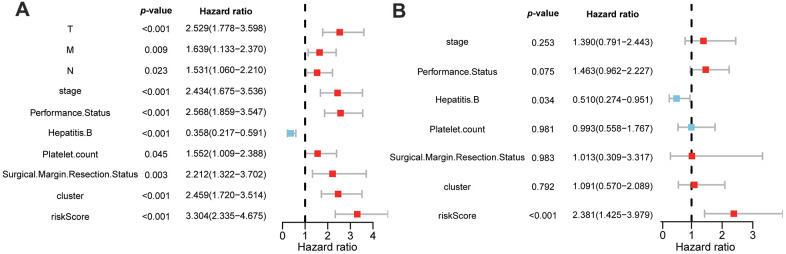
**Univariable and multivariable Cox regression analyses of clinicopathological characteristics and GMPM for HCC patient’s prognosis.** Univariable analyses of clinicopathological features and GMPM in TCGA (**A**). Multivariable analyses of clinicopathological features and GMPM in TCGA (**B**). GMPM, glycolysis-associated multi-omics prognostic model.

To investigate whether the GMPM risk score was an independent risk factor for HCC prognosis prediction, we conducted univariable and multivariable Cox regression analyses. The results of the univariable analysis showed a significant hazard ratio of tumor stage, ECOG performance status, HBV infection status, platelet count, surgical margin status in resection, cluster and riskscore in the OS of patients with HCC (*p* < 0.05; Figure 7A). Furthermore, multivariable analysis demonstrated that GMPM and HBV infection status was an independent predictive factor of OS in HCC (*p* < 0.001; Figure 7B). These results further confirmed the prognostic potential of the GMPM signature in predicting OS in patients with HCC.

## DISCUSSION

In this study, we established subtype-stratification models based on multiomics glycolytic-associated gene status. We stratified patients with HCC into Glycolysis-H, Glycolysis-M, and Glycolysis-L subgroups, which were associated with different expression patterns based on glycolysis-related genes. Further, the Glycolysis-H+M subgroup presented with a significantly worse OS than its counterpart, and this result is consistent with those of previous studies [11]. Notably, the Glycolysis-H+M subgroup had a higher T-stage and pathological grade. Thus, it may be speculated that undifferentiated tumors with high T-stage may possess distinct glycolysis-associated gene expression patterns that possibly exacerbate tumor progression and promote poor prognosis.

The degree of malignancy in HCC is inevitably correlated with energy metabolism, as the liver vitally acts as the coordinator of maintaining energy metabolism homeostasis. Metabolism reprogramming is closely correlated with tumorigenesis. Our GSEA-based KEGG enrichment analysis also revealed pathways previously been identified as abnormal signaling pathways in HCC [12]. In fact, Warburg effect is characterized in many malignancies by the shift of the glycometabolic pathway from oxidative phosphorylation to aerobic glycolysis in mitochondria even with a sufficient supply of oxygen. Although this shift lowers the efficacy of ATP supply, it also reduces the dependence of tumor cells on oxygen [13]. Further, its intermediate metabolites also support the protein, lipid, and nucleic acid synthesis pathways and pentose phosphate pathway of tumors. Such metabolism also supports the rapid proliferation and metabolism of tumor cells [14]. Thus, the activity of the glycolysis pathway may be closely linked to the malignancy of tumors and further affect the survival of patients, especially in patients with HCC. In such cases, predictive models can help us understand the underlying mechanisms of HCC and improve prognosis prediction of individual patients.

To further investigate the differences among the subgroups, a multiomics analysis was carried out. Although liver cancer stem cells account for a small proportion of all tumor cell types, they maintain tumorigenic and metastatic properties of the tumor by their genetic and epigenetic factors [15, 16]. In our study, high mRNAsi index in the Glycolysis-H+M subgroup suggested that the glycolysis-related gene expression pattern with poor prognosis had higher tumor stemness, and this may have further resulted in poor patient prognosis. Stem cell characteristics acquisition strongly influences tumor progression [17], which is in accordance with the increase in mRNAsi during progression as previously described [10]. To the best of our knowledge, this study is the first to analyze the correlation between stemness characteristics of cancer and glycolysis-related gene expression.

Tumor microenvironment (TME) contributes largely to the development of tumor heterogeneity and the development of tumors [18]. In the TME, immune infiltration patterns are valuable for prognostic assessment in HCC [19]. The interdependent relationship between tumor metabolism and immune infiltration has been reported in previous studies as well [20]. Our results showed significantly elevated B cells, CD4+ and CD8+ T cells, neutrophils, macrophages, and dendritic cells in the Glycolysis-H+M subgroup than in Glycolysis-L subgroup; this indicates that the immune patterns tend to be closely related to the glycolytic state of a tumor. This result was consistent with previous reports [21], suggesting that these subgroups of glycolysis could be used to preliminarily evaluate the immune characteristics of cancer. Furthermore, the use of immunotherapy in the Glycolysis-H+M subgroup may be more effective.

In addition, investigation of the somatic mutation landscape among the glycolysis subgroups revealed significantly higher *TP53* and *RB1* mutational burdens in the Glycolysis-H+M subgroup. *TP53* and *RB1* are believed to be tumor-suppressor genes in multiple tumors [22, 23]. In fact, *TP53* has the highest mutation frequency in HCC, and its functional loss enables survival of DNA-damaged cells and escape of apoptosis, thereby affecting the progression of HCC patients [22]. Moreover, *RB1* is a known tumor-suppressor gene [24], and the loss of *RB1* is the main mechanism of acquired resistance in HCC [23].

In recent years, glycolysis-related genes have caused widespread concern for its association with tumors. Jiang et al. first identified and validated a six-mRNA glycolysis-related signature for predicting outcomes for patients with HCC [21]. However, they only analyzed gene expression profiles in their study. To make our prediction model more robust, we conducted a multiomics analysis for glycolysis-related genes, and included CNVs, gene mutations, and gene expression levels to build a novel, glycolysis-associated predictive model for HCC. Consequently, our GMPM had a stable and steady prediction accuracy at 1-, 3-, and 5-years with AUC values above 0.745 for all. Thus, this model may provide a more efficient way of screening high-risk patients with HCC associated with poor prognosis. Further, the genes included in the GMPM have been correlated with tumorigenesis and aggressiveness of HCC. For instance, *ANG* plays a critical role in angiogenesis in HCC, and is involved in the regulation of immune response [25]. *CLDN9* is an HCC proto-oncogene that increases the invasiveness and migration capability of cancer cells by affecting the STAT3 signaling pathway [26]. *G6PC* is a hepatocyte terminal differentiation marker, and Yan et al. demonstrated that the overexpression of *GPR87* could upregulate CD133 expression to promote tumor initiation [27]. High expression of *SOX9* is closely related to the poor prognosis in patients with HCC [28]. Elevated expression of *TFF3* has been found in HCC, and it is associated with poor patient survival outcomes and clinical features [29]. *ADH* is known to be repressed in the HepG2 human hepatoma cell line [30]. Furthermore, some genes were associated with glucose metabolism as well. *GCK* plays a vital role in liver and pancreatic beta cells to regulate glucose distribution and synthesis, and its activity is competitively inhibited by *GCKR* [31]. Chen et al. found significant downregulation of *GYS2* through the HBx/GYS2/p53 pathway in HCC, which results in the deregulation of glycogen metabolism [32]. *PCK* is a protein in the hepatic gluconeogenesis pathway whose low expression is associated with poor prognosis in patients with HCC [33]. Thus, our strategy screened out glycolysis-related gene panels with robust prognosis predictive ability, and may guide the investigation of underlying mechanisms in glycolysis-associated HCC tumorigenesis. Although this study provides a new perspective of glycolysis-related genes in molecular subtyping and prognostic prediction of HCC based on integrative multiomics analyses, its limitations must be acknowledged. First, the prognostic value of our risk stratification model needs to be validated in more patients with HCC with multicentered data to further increase its credibility. Second, the molecular understanding of glycolysis-associated genes used in the risk stratification model needs further investigation with functional experiments to unravel the role of glycolysis in HCC.

## CONCLUSIONS

To conclude, by stratifying the patients with HCC with glycolysis-associated genes, we identified different prognostic, clinical, and immune features between patients with different glycolysis patterns. Consequently, we used this novel approach to identify key glycolysis-related genes by developing a GMPM, which presented as an independent risk factor with robust prognostic predictive ability for HCC. Taken together, this study provides a guide for the clinical management of patients with HCC, and genes related to our model may provide promising targets for HCC treatment.

## MATERIALS AND METHODS

### Data acquisition and preprocessing

Transcriptome profiles, masked somatic mutation data, CNV files, and clinicopathological information of 374 patients with HCC were acquired from TCGA-liver hepatocellular carcinoma (LIHC) program in TCGA (https://portal.gdc.cancer.gov/). Additionally, we obtained gene expression files and clinicopathological data of 232 patients with HCC from the LIRI-JP project in the International Cancer Genomics Consortium (ICGC; https://icgc.org/) database. The gene expression data were normalized and log2-transformed for subsequent analyses. The samples that lacked important clinicopathological or survival information were excluded from further analyses.

### Clustering analysis of glycolysis-associated genes

Genes involved in glycolysis and glycolysis-related signaling pathways were downloaded via the Kyoto Encyclopedia of Genes and Genomes (KEGG) from Gene Set Enrichment Analysis (GSEA; http://software.broadinstitute.org/gsea/index.jsp). Based on the expression levels of the glycolysis-associated genes, patients with HCC from TCGA and ICGC were stratified into different subgroups using hierarchical clustering analysis. Furthermore, Kaplan-Meier method was employed to analyze and compare the overall survival (OS) in the different glycolysis-associated subgroups.

### Evaluation of cancer stem cells characteristics

To calculate the mRNA expression and DNA methylation status-based stemness indices of the samples, a predictive model, previously reported by one-class logistic regression (OCLR), was adopted. OCLR was calculated using pluripotent stem cell samples from the Progenitor Cell Biology Consortium dataset [10]. The stemness indices ranged from low (zero) to high (one) to scale the stemness features of the samples. The differences in stemness indices between different glycolysis subgroups were analyzed using Wilcoxon rank-sum test. Further, we conducted a correlation analysis between the expression levels of the glycolysis-associated genes and stemness indices. Genes with an absolute value of correlation coefficient r > 0.4 and p < 0.001 were plotted using Cytoscape [34] (Version:3.8.1, https://cytoscape.org/).

### Analysis of immune infiltration

CIBERSORT algorithm was used to calculate the relative abundance of 22 immune cell types according to a previously reported procedure [35]. The proportion of each immune cell was evaluated and compared across the groups using Wilcoxon rank-sum test. Similarly, we conducted a correlation analysis between the glycolysis-associated gene expression levels and immune cell abundance. The correlations with |*r*| > 0.4 and *p* < 0.001 were plotted using Cytoscape.

### Multiomics analysis of different glycolysis-associated subgroups

Mutation data of patients with HCC from TCGA were analyzed and visualized using maftools package in R software [36]. Genes with the highest tumor mutation frequencies and their percentages in different glycolysis-associated subgroups were displayed using a waterfall plot. Chi-square test was employed to screen significantly different CNVs (adjusted *p* < 0.05) among the different glycolysis-associated subgroups.

Using RNA-sequencing data of patients with HCC in TCGA, we compared the expression level of each gene among the different glycolysis-associated subgroups using the limma R package [37]. A false discovery rate (FDR) < 0.05 and |log_2_fold change (FC) |> 1 was set as the threshold to identify differentially expressed genes (DEGs). GSEA was carried out to identify significantly upregulated and downregulated KEGG pathways among the glycolysis subgroups with an FDR < 0.05 [38, 39].

### Identification and analysis of different CNV status genes among glycolysis subgroups based on multiomics data

Genes with different expression levels and CNV statuses were obtained by taking the intersection of the DEGs and genes with different CNV statuses among the different glycolysis-associated subgroups. These genes were subjected to gene ontology (GO) and pathway analyses. For the GO analysis, we identified enriched terms related to biological processes (BP), molecular functions (MF), and cell components (CC). Terms with FDR < 0.05 in GO and pathway analyses were visualized [40]. Furthermore, we conducted a univariable Cox regression analysis to evaluate the correlation of the expression levels of these genes and OS of the patients with HCC.

### Construction and validation of a glycolysis-associated prognostic model

A least absolute shrinkage and selection operator (LASSO) regression model was used to extract key glycolysis-associated genes based on the DEGs and CNV statuses to construct a glycolysis-associated multiomics prognostic model (GMPM) using glmnet R package [41]. Next, the risk score of each patient with HCC in TCGA and ICGC was calculated by adding the multiplication of each glycolysis-associated gene expression level with its corresponding regression coefficients. Correlation analysis between key glycolysis-associated genes was further conducted.

Using the median value of the GMPM risk score in TCGA as the cutoff value, patients with HCC (in both TCGA and ICGC cohorts) were stratified into high- and low-risk subgroups, separately. The OS of patients was calculated and compared using the Kaplan-Meier method, and the heatmap of genes used in the GMPM was plotted. Finally, univariable and multivariable Cox regression analyses were conducted to identify independent prognostic predictors associated with the OS of patients with HCC in TCGA.

## Supplementary Material

Supplementary Figures

Supplementary Table 1
