# Supplementary material for: Identification and validation of a glycolysis-associated multiomics prognostic model for hepatocellular carcinoma
Source: Aging (Albany NY). 2021 Mar 3;13(5):7481–98. doi: 10.18632/aging.202613 (PMC7993684; doi:10.18632/aging.202613)
Supplement: Supplementary Figures [file aging-13-202613-s001.pdf]

SUPPLEMENTARY FIGURES

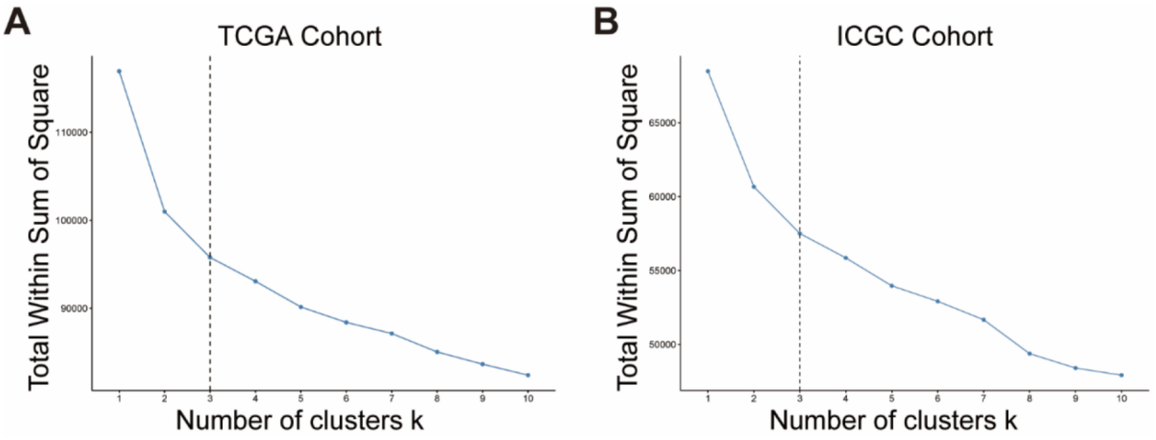

Supplementary Figure 1. Elbow plot of TCGA (**A**) and ICGC (**B**) cohort. Vertical axis showed the total within sum of square, horizontal axis represented the number of clusters.

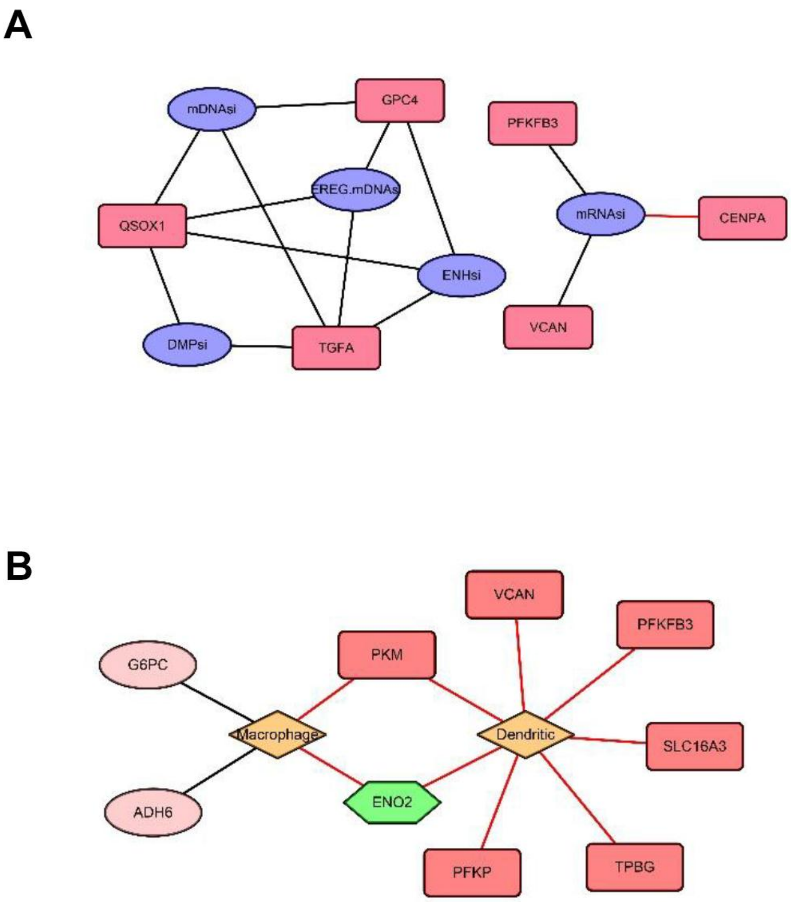

Supplementary Figure 2. (**A**) The regulatory network of stem cell indices and glycolysis-associated genes. (**B**) The regulatory network of macrophages, dendritic cells, and glycolysis-associated genes. Black edge for negative-regulated and red edge for positive-regulated.

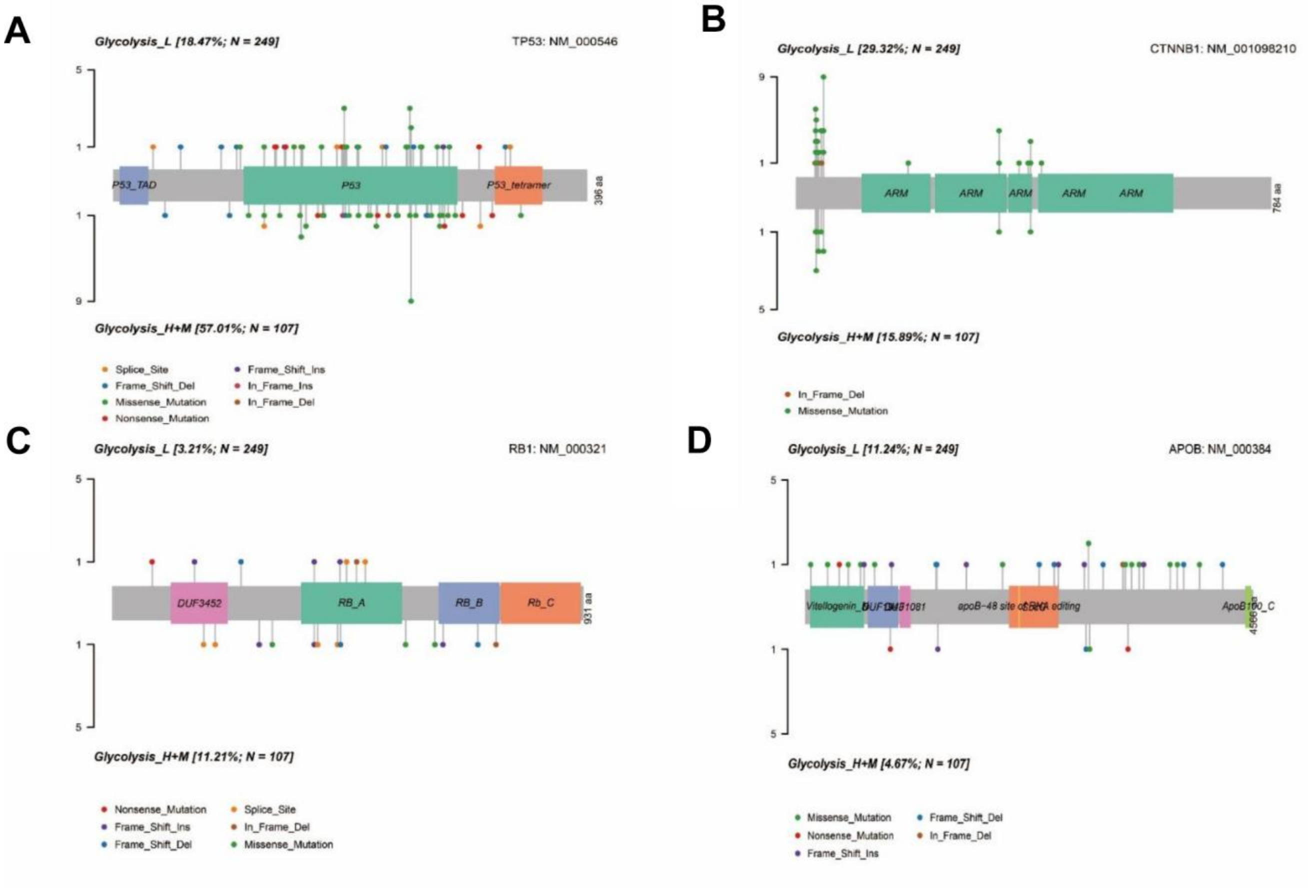

Supplementary Figure 3. The lollipop plot of TP53 (**A**), CTNNB1 (**B**), RB1 (**C**), and APOB (**D**) showed each mutation with its location and accumulated burden of each gene.
