# Supplementary material for: Identification and validation of a glycolysis-associated multiomics prognostic model for hepatocellular carcinoma
Source: Aging (Albany NY). 2021 Mar 3;13(5):7481–98. doi: 10.18632/aging.202613 (PMC7993684; doi:10.18632/aging.202613)
Supplement: Supplementary Table 1 [file aging-13-202613-s002.pdf]

## SUPPLEMENTARY TABLE

**Supplementary Table 1. Glycolysis-associated genes from KEGG signaling pathways.**

| | | | | | | | | | | | |
|---|---|---|---|---|---|---|---|---|---|---|---|
| AAAS | ALDH3B2 | CACNA1H | DDIT4 | GAL3ST1 | GUSB | LDHAL6A | NUP133 | PCK1 | PKP2 | RBCK1 | TGFBI |
| ABCB6 | ALDH7A1 | CAPN5 | DEPDC1 | GALE | GYS1 | LDHAL6B | NUP153 | PCK2 | PLOD1 | RPE | TKTL1 |
| AC010618.1 | ALDH9A1 | CASP6 | DLAT | GALK1 | GYS2 | LDHB | NUP155 | PDHA1 | PLOD2 | RRAGD | TPBG |
| AC074143.1 | ALDOA | CD4 | DLD | GALK2 | HAX1 | LDHC | NUP160 | PDHA2 | PMM2 | SAP30 | TPI1 |
| ACSS1 | ALDOB | CD44 | DPYSL4 | GALM | HDLBP | LHPP | NUP188 | PDHB | POLR3K | SDC1 | TPR |
| ADH1A | ALDOC | CDK1 | DSC2 | GAPDH | HK1 | LHX9 | NUP205 | PDK3 | POM121 | SDC2 | TPST1 |
| ADH1B | ALG1 | CENPA | ECD | GAPDHS | HK2 | MDH1 | NUP210 | PFKFB1 | POM121C | SDC3 | TSTA3 |
| ADH1C | ANG | CHPF | EFNA3 | GCK | HK3 | MDH2 | NUP214 | PFKFB2 | PPFIA4 | SDHC | TXN |
| ADH4 | ANGPTL4 | CHPF2 | EGFR | GCKR | HMMR | ME1 | NUP35 | PFKFB3 | PPIA | SEC13 | UGP2 |
| ADH5 | ANKZF1 | CHST1 | EGLN3 | GCLC | HOMER1 | ME2 | NUP37 | PFKFB4 | PPP2CA | SEH1L | VCAN |
| ADH6 | ARPP19 | CHST12 | ELF3 | GFPT1 | HS2ST1 | MED24 | NUP43 | PFKL | PPP2CB | SLC16A3 | VEGFA |
| ADH7 | ARTN | CHST2 | ENO1 | GLCE | HS6ST2 | MERTK | NUP50 | PFKM | PPP2R1A | SLC25A10 | VLDLR |
| ADORA2B | AURKA | CHST4 | ENO2 | GLRX | HSPA5 | MET | NUP54 | PFKP | PPP2R1B | SLC25A13 | XYLT2 |
| ADPGK | B3GALT6 | CHST6 | ENO3 | GMPPA | IDH1 | MIF | NUP58 | PGAM1 | PPP2R5D | SLC35A3 | ZNF292 |
| AGL | B3GAT1 | CITED2 | ERO1A | GMPPB | IDUA | MIOX | NUP62 | PGAM2 | PRKACA | SLC37A4 | ALDH3B1 |
| AGRN | B3GAT3 | CLDN3 | EXT1 | GNE | IER3 | MPI | NUP85 | PGAM4 | PRKACB | SOD1 | BPNT1 |
| AK3 | B3GNT3 | CLDN9 | EXT2 | GNPDA1 | IGFBP3 | MXI1 | NUP88 | PGK1 | PRKACG | SOX9 | DCN |
| AK4 | B4GALT1 | CLN6 | FAM162A | GNPDA2 | IL13RA1 | NANP | NUP93 | PGK2 | PRPS1 | SPAG4 | G6PD |
| AKR1A1 | B4GALT2 | COG2 | FBP1 | GOT1 | IRS2 | NASP | NUP98 | PGM1 | PSMC4 | SRD5A3 | GPR87 |
| ALDH1A3 | B4GALT4 | COL5A1 | FBP2 | GOT2 | ISG20 | NDC1 | NUPL2 | PGM2 | PYGB | STC1 | LDHA |
| ALDH1B1 | B4GALT7 | COPB2 | FKBP4 | GPC1 | KDELR3 | NDST3 | P4HA1 | PGM2L1 | PYGL | STC2 | NUP107 |
| ALDH2 | BID | CTH | FUT8 | GPC3 | KIF20A | NDUFV3 | P4HA2 | PGP | QSOX1 | STMN1 | PC |
| ALDH3A1 | BIK | CXCR4 | G6PC | GPC4 | KIF2A | NSDHL | PAM | PHKA2 | RAE1 | TALDO1 | PKM |
| ALDH3A2 | BPGM | CYB5A | G6PC2 | GPI | LCT | NT5E | PAXIP1 | PKLR | RANBP2 | TFF3 | TGFA |
